# Household Water, Sanitation, and Hygiene Practices Impact Pathogen Exposure in Remote, Rural, Unpiped Communities

**DOI:** 10.1089/ees.2020.0283

**Published:** 2021-05-24

**Authors:** Kaitlin J. Mattos, Laura Eichelberger, John Warren, Aaron Dotson, Millie Hawley, Karl G. Linden

**Affiliations:** ^1^ Civil, Environmental, and Architectural Engineering Department, the Mortenson Center in Global Engineering, University of Colorado, Boulder, Colorado, USA.; ^2^ Division of Environmental Health and Engineering, Alaska Native Tribal Health Consortium, Anchorage, Alaska, USA.; ^3^ Civil Engineering Department, University of Alaska Anchorage, Anchorage, Alaska, USA.; ^4^ Native Village of Kivalina, Kivalina, Alaska, USA.

**Keywords:** pathogen exposure, remote, rural, unpiped, WASH

## Abstract

Household water, sanitation and hygiene (WASH) practices in remote, rural, and unpiped communities are likely to impact exposure to pathogens beyond the fecal–oral transmission routes that are typically prioritized in WASH interventions. We studied 43 homes in two remote, rural, unpiped communities in Alaska to evaluate seasonal water haul, water sources, water quality, and water reuse, as well as greywater and human waste disposal over 1 year. Hauled quantities of water reportedly ranged from 3.0 to 5.4 gallons per capita per day (gpcd) depending on the community and season. Natural, untreated water sources contributed 0.5–1.1 gpcd to household water availability. Reported quantities of water hauled were significantly correlated with total water storage capacity in the home. Total coliforms were detected in 30–60% of stored household water samples from treated and untreated sources, and total coliform counts were significantly higher in specific sources and during specific seasons. Exposure to pathogens during periods of low water access, from untreated water reuse, from greywater disposal and from human waste disposal are important pathways of disease transmission in these remote, rural, unpiped communities. We discuss intermediate steps that can be taken at the household and community levels to interrupt exposure pathways before piped infrastructure is installed. This model of examining specific household practices to determine transmission routes can be applied to other remote communities or unique conditions to aid in the recommendation of targeted WASH interventions.

## Introduction

### The role of WASH in reducing pathogen exposures

The international Sustainable Development Goal of safe water and safely managed sanitation aims to support health and well-being for all (Rosa, 2017), but specifically targets poor, marginalized, and disadvantaged populations (United Nations General Assembly, 2010). This aim presents specific challenges to remote, rural communities where residents experience a lack of water and sanitation infrastructure (Wagner and Lanoix, 1958), but where resources, conditions, and practices may differ drastically from more heavily populated and well-characterized urban areas (World Health Organization Joint Monitoring Program, 2017). Specific conditions in remote and rural communities may have an impact on how diseases are spread and thus on how health and wellbeing can best be protected through water, sanitation, and hygiene (WASH) interventions.

WASH interventions improve health by disrupting the exposure of individuals to pathogens through consumption, inhalation, and contact (World Health Organization, 2011; Prüss-Ustün *et al.*, 2014). Typically, water interventions focus on removing fecal pathogens and improving water quality through treatment and storage (Sobsey, 2002; World Health Organization, 2011) and ensuring sufficient quantities of water are available to remove the pathogens that individuals do come into contact with (Howard and Bartram, 2003; Mara *et al.*, 2003). Sanitation interventions attempt to contain and treat human waste to prevent human contact with fecal pathogens (Mara *et al.*, 2010; Stenström, 2011; World Health Organization, 2018). The f-diagram, which shows exposure routes from human feces to new hosts through various intermediate carriers, is a useful tool for looking at disruptions that can be made in the transmission of fecal–orally transmitted diseases through sanitation, water treatment, and hygiene practices (Bateman, 1994). However, WASH is also critical in preventing the transmission of other classes of communicable diseases, such as water-washed diseases, foodborne diseases, and airborne-transmitted infections (Webber, 2004). Improved models can be created by incorporating knowledge of household WASH practices and describing the exposure pathways specific to an area or situation to determine where transmission of disease can be interrupted (e.g., in Hurd *et al.*, 2017; Robb *et al.*, 2017; Navab-Daneshmand *et al.*, 2018).

### Specific WASH challenges in remote and rural Alaska Native communities

The United States is considered to have 100% coverage of safely managed water and sanitation services across the country (World Health Organization Joint Monitoring Program, 2015), but the state of Alaska still has an estimated 6% of its population state-wide living without access to these services, in approximately 30 communities. Many more communities experience seasonal outages of services or have aging infrastructure. Meanwhile, the gap between infrastructure funding needs and funding available is growing annually (Alaska Department of Environmental Conservation, 2017); so many communities are not likely to have their infrastructure needs addressed in the near future. Most of the unpiped or partially piped communities in Alaska comprise Alaskans of Indigenous descent (Alaska Native peoples) living in rural and remote areas with <300 residents per community that are off the road system, thus difficult to access (Alaska Department of Labor and Workforce Development, 2010). As an additional challenge, utility costs (as a percent of household income) are much higher in remote Alaskan communities than those on the state's limited road system (Colt *et al*., 2003). As a result, many households must allocate limited resources to food, energy, and water expenses (Eichelberger, 2010).

As a result of the lack of piped infrastructure, Alaska Native residents in unpiped villages conserve water used in the home, using very little water for washing hands, clothes, dishes, and for cleaning purposes, or reusing water multiple times for different hygiene activities (Eichelberger, 2010; Thomas *et al.*, 2016; Hickel *et al.*, 2017). Bucket latrines or pit latrines are used for human waste. Greywater, urine, and feces are either disposed of in pit latrines, collected in community hoppers, and hauled to open sewage lagoons, or disposed of on the ground near the home. All communities have a central watering point available (often for a fee) for self-hauling treated water to the home, and some communities have a central facility called a washeteria that provides access to showers and laundry machines. Water at these facilities is treated using filtration and chlorine disinfection. Water plants are usually operated by a single local operator with engineering support from regional and state offices. Some households supplement treated water with self-hauled water from untreated natural sources such as rain catchment systems, rivers, or springs (Ritter *et al.*, 2014; Eichelberger, 2017).

A lack of household WASH infrastructure has been associated with high rates of skin and respiratory infections in villages without piped water (Gessner, 2008; Hennessy *et al.*, 2008; Thomas *et al.*, 2016). Lack of access to sufficient quantities of clean water and safely managed sanitation is likely to influence not only infectious disease incidence, but also stress levels and overall wellbeing in unpiped communities (Eichelberger, 2016, 2017). However, most of the household practices associated with the lack of WASH infrastructure access are not understood by funding agencies and engineers who are in the best position to serve unmet needs.

To efficiently improve health in challenging WASH contexts, pathways of pathogen exposure related to household WASH practices need to be explored, described, and considered in the provision of services. This study systematically collected information on water haul practices, water sources used, water quality, water reuse, greywater disposal, and human waste disposal in unpiped households in two remote Alaska Native communities over four seasons. Although a few studies have quantified in-home treated water use in unpiped Alaskan communities (e.g., Eichelberger, 2010, 2017; Thomas *et al.*, 2016), to our knowledge, this study is the first to collect seasonal data on household water use, microbial quality from different water sources, and waste management practices in remote Alaska. We use the data collected here to estimate deficiencies in meeting health-related water use requirements and to model pathways of exposure risk. This work highlights overlooked gaps in water and sanitation interventions and can assist planners, engineers, and public health practitioners in designing infrastructure and supporting programs that will address specific needs for remote communities.

## Methods and Protocols

### Study communities and engagement

This study was conducted with two Alaska Native communities located off the road system in rural Alaska. The communities were approached because (1) they did not have piped water and sanitation systems and (2) they had an on-going water and sanitation improvement project with the Alaska Native Tribal Health Consortium (ANTHC). Researchers obtained permission and approvals from tribal councils, regional health organizations, the ANTHC research review committee, and the institutional review boards (IRB) of the Alaska Area (#2018-03-009) and University of Colorado (#18-0384). Tribal councils and community advisory committees have approved dissemination of the results reported here.

### Household recruitment

Researchers recruited households to participate in the study if they did not have piped water or sanitation in their home and if they were interested in having researchers visit their home seasonally to talk with them about water and sanitation and collect water samples from their household water storage containers. Researchers employed a snowball recruiting technique. The project was announced at community meetings and on the community VHF (very high frequency) radio, and sign-up sheets were circulated to tribal and city staff, at community gathering places, and throughout the community to get contact information for interested households. When households agreed to participate, researchers asked if they knew of others in the community who would be interested. Participation was not tied to receipt of health care or water and sanitation services from ANTHC. On the first visit, researchers described the goals and activities of the research project and obtained a signed consent form from the head(s) of household for participation in the study. We present aggregated and anonymous data here to protect the identity of individuals. Community names have been withheld until the communities choose to be identified.

### Household interviews

#### Data collection

Researchers visited households four times over the course of 1 year in winter, spring, summer, and fall (October 2018–September 2019). Visit dates were chosen based on the instruction of community advisors. We attempted to select dates that represented typical weather for that season and that maximized time in between visits.

At each household visit, the same researcher (author K.J.M.) conducted a semistructured interview with the head(s) of household about household and community water and waste management practices. She recorded the age and gender of the respondent. Interviewees were asked how many residents lived in the home, what sources of water they used, how much water they hauled, why and how they reused water, and how they disposed off greywater and wastewater. Interviewees were shown a reference page of water container sizes to assist with estimation of volumes, and detailed notes were taken about specific practices. All questions were prompted by asking specifically about average practices in the current season. Follow-up questions were asked to try to improve accuracy of average frequencies and volumes for each set of practices. Interviewees were also asked about house ownership, vehicle ownership, and number of high school graduates in the home as indicators of socioeconomic status.

#### Data analysis

Responses to interview questions about water haul from each were converted into average gallons of water per capita per day (gpcd) by taking average values from ranges provided and dividing them by total number of residents in the home. Water from all sources was considered “hauled,” even from onsite rainwater and snow collection, because of the effort required to get the water into the home for use. Pearson's correlation tests were performed on log-transformed total household residents versus log-transformed total gpcd hauled and on log-transformed total household storage volume versus log-transformed total gpcd hauled. A Spearman's rank correlation test was performed on number of vehicles owned by a household versus total gpcd hauled. Greywater disposal responses from interviews were categorized as ≤5 m from the home on the ground surface, ≥5 m from the home on the ground surface, or underground (in a hole or pit latrine). For homes using bucket latrines, the average number of bag-days (number of plastic bags of human waste × number of days) that human waste sat outside before final disposal was calculated based on the frequency of emptying the toilet and the frequency of hauling waste away from the home.

### Water quality sampling

#### Data collection

At each home, water quality samples were taken from each type of water storage container of each water source in sterile 100 mL bottles containing sodium thiosulfate to neutralize any residual chlorine. Samples were drawn using the same practices that household members typically use; therefore, spouts were not sterilized before sampling and the same water dippers used by the household were used during sampling. Samples were only taken from household stored water, and not from wash basin reuse water or greywater containers. Data were recorded on the source of water in the container, the size and type of container, and whether the container was covered at the time of sampling. Samples were analyzed at a field laboratory in the community within 12 h of sampling. Microbiological analysis was conducted using Colilert^®^ and Quanti-tray^®^/2000 products from IDEXX (Westbrook, ME) to yield the most probable number of total coliforms or *Escherichia coli* per 100 mL of sample (MPN/100 mL). The test range is 1–1011.1 MPN/100 mL without dilution. No dilutions were carried out owing to field laboratory equipment limitations. Samples were incubated at 35°C for 24 h.

#### Data analysis

Average total coliforms and *E. coli* values (MPN/100 mL) were calculated by season and source of water. Samples that tested below the limit of detection (≤1 MPN/100 mL) were considered to be absent of coliform bacteria, and these values were compared with the US Environmental Protection Agency's primary water quality standard of 0 MPN/100 mL (US Environmental Protection Agency, 2009). Wilcoxon rank sum tests were conducted to determine differences in total coliform counts between water from various sources, samples across seasons, water stored in covered or uncovered containers, and the same water source from a container before filtration versus after filtration from a dispenser or filtered pitcher. Nondetect samples (≤1 MPN/100 mL) were attributed a value of “1,” and samples at the quantification limit (≥1011.1 MPN/100 mL) were attributed a value of “1011.1” for statistical analyses, including Wilcoxon tests. The data were not transformed for these analyses.

## Results

### Respondents

#### Communities

One community in Interior Alaska and one community in Northwest Alaska participated in the study. Both communities are located off the road system and are primarily reached by airplane, by overland travel by snow-machine in winter, and with boat and barge access by river or ocean in summer. Both communities rely heavily on subsistence economies for their livelihoods and have ∼30% of their population living below the poverty line (State of Alaska Department of Labor, 2016). Most wage jobs available in the communities are at the tribal, city, or regional corporation offices, the school, or the water plant and washeteria. Some residents may also be seasonal or shift-workers at nearby mining or oil operations or on construction or firefighting crews. Community life is centered around seasonal fishing, hunting, and gathering activities and daily tasks of hauling fuel (firewood, stove oil, gasoline) and water, childcare, and homemaking.

The Interior community has a population of ∼200. Of ∼37 occupied households in the community, an estimated 32 were approached and 19 chose to participate for at least one season (51% participation), representing 29% of the population. Over 70 h of interviews were completed in the Interior over four seasons in January, April, July, and September 2019. The Northwest community has a population of ∼400. Of ∼60 occupied households in the community, an estimated 42 were approached and 24 chose to participate (40% participation), representing 31% of the population. Over 100 h of interview were completed over four seasons in October 2018, and February, May, and July 2019.

#### Households and interviewees

Average household size was 2.9 residents in the Interior with an average of 0.8 children under 18 per home, and 5.2 residents in the Northwest community with an average of 2.2 children under 18 per home. Approximately two thirds of participating households owned their homes. In the Interior, 93% of adults in participating homes had graduated high school, compared with 67% in the Northwest. The Interior also had more households that either owned or had regular access to a functioning vehicle (84%) compared with the Northwest (54%; Table 1).

**Table 1.** Population Characteristics from Two Study Communities— One in the Interior and One in the Northwest Region of Alaska

Study population characteristics	Interior community	Northwest community	Total
Number of HH in study (% of total HH)	19 (51%)	24 (40%)	43 (44%)
Total no. of HH in community	37	60	97
Average no. of persons per home	2.9	5.2	4.2
Average no. of children per home	0.8	2.2	1.6
Adults in study HH who graduated high school/total no. of adults in study HH, *n* (%)	27/29 (93)	42/63 (67)	69/92 (75)
HH who own home, *n* (%)	12 (63)	16 (67)	28 (65)
HH with access to functioning vehicle, *n* (%)	16 (84)	13 (54)	29 (67)
Median age of respondent	47	55	54
female respondents, *n* (%)	14 (58)	18 (53)	32 (55)

HH, household.

### Water quantity

#### Household water quantity by season

Reported quantities of water hauled and used in the household varied by household, season, and community. Mean reported water quantity hauled from all sources ranged from 3.0 to 5.4 gpcd depending on season and community (Table 2). Mean water storage capacity in the home ±1 standard deviation (SD) was 19 ± 22 gallons/person (range = 2–108) in the Interior and 29 ± 44 gallons/person (range = 2–243) in the Northwest. In both communities, the majority of homes had <100 gallons of total storage capacity (Interior: *n* = 13 homes, 81%; Northwest: *n* = 19 homes, 79%). Storage capacity expanded in the summer and fall in the Interior (mean household water storage volumes: fall = 66.3 gal, winter = 28.2 gal, spring = 27.9 gal, summer = 81.1 gal) and in the summer in the Northwest (mean household water storage volumes: fall = 91.4 gal, winter = 95.5 gal, spring = 96.3 gal, summer = 142.4 gal), likely because people bought new or repurposed existing containers that had not previously been storing water to accommodate rainwater availability. There was no correlation between total number of residents and gpcd hauled (*p* = 0.3) or between number of vehicles owned and gpcd hauled (*p* = 0.2 across all seasons, *p* = 0.7, 0.7, 0.8, and 0.4 in fall, winter, spring, and summer, respectively). There was a significant correlation between water storage capacity in the home and total gpcd hauled (*p* = 0.0003).

**Table 2.** Reported Quantity of Water Hauled to the Home in Gallons Per Capita Per Day and as a Percent of the WHO Recommendation for Intermediate Water Access (13.2 gpcd, Howard and Bartram 2003)

	Interior gpcd (mean ± SD)	Northwest gpcd (mean ± SD)	Total gpcd (mean ± SD)	Total water hauled as % of WHO rec
Water hauled to the home–all sources				
Fall	3.3 ± 4.9 (*N* = 11)	4.4 ± 5.8 (*N* = 22)	4.0 ± 5.4 (*N* = 33)	30%
Winter	3.5 ± 3.2 (*N* = 12)	3.9 ± 4.1 (*N* = 22)	3.8 ± 3.7 (*N* = 34)	29%
Spring	5.4 ± 3.1 (*N* = 11)	3.4 ± 3.5 (*N* = 23)	3.9 ± 3.4 (*N* = 34)	30%
Summer	3.0 ± 1.8 (*N* = 13)	5.1 ± 6.6 (*N* = 23)	4.3 ± 5.5 (*N* = 36)	37%
Water hauled to the home–treated watering point (WTP) only				
Fall	1.9 ± 1.6 (*N* = 11)	4.4 ± 5.8 (*N* = 21)	3.5 ± 4.9 (*N* = 32)	27%
Winter	2.7 ± 1.9 (*N* = 12)	3.9 ± 4.0 (*N* = 21)	3.6 ± 3.5 (*N* = 33)	27%
Spring	3.5 ± 2.0 (*N* = 11)	3.4 ± 3.5 (*N* = 23)	3.4 ± 3.0 (*N* = 34)	26%
Summer	2.2 ± 1.2 (*N* = 13)	3.5 ± 3.2 (*N* = 23)	3.0 ± 2.7 (*N* = 36)	23%

“All sources” includes treated community watering point (wtp), store-bought bottled water, rainwater, snow melt, and river water.

SD, standard deviation; WTP, water treatment plant. *N* refers to number of households with data available for a given season.

#### Household water sources

**FIG. 1.**
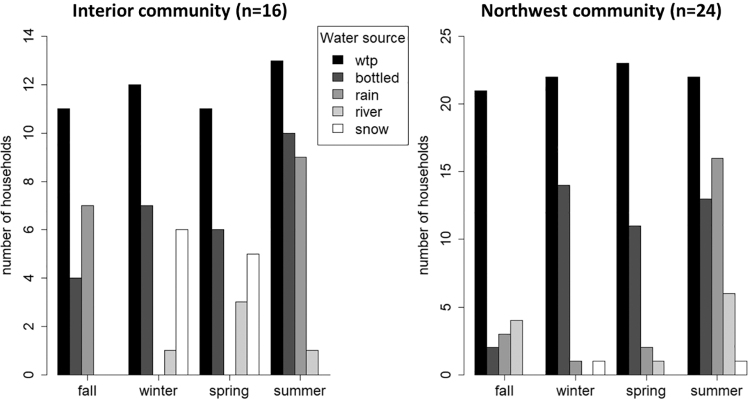
Water sources used in the home varied by season and differed between the two communities. “WTP” refers to the community's watering point at the centralized water treatment plant.

**FIG. 2.**
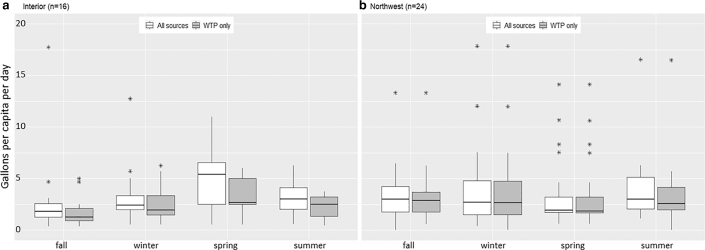
Variation in household water haul by community and season, considering all water sources versus community water treatment plant (“WTP”) only. Boxes represent interquartile range (IQR). Horizontal mid-line represents median value. Vertical lines show values less than 1.5 times the IQR. Asterisks denote outliers outside of 1.5 times the IQR above the upper quartile and below the lower quartile.

Across all seasons, all participating households reported using water from the community's centralized treated watering point at the water treatment plant (WTP) as their primary water source, except for one home that did not use WTP water in the fall or summer. However, almost all participants (Interior: *n* = 16, 100%; Northwest: *n* = 22, 92%) reported supplementing WTP water with natural, untreated water sources such as river water, snow melt, or rainwater and with bottled water (Fig. 1) in at least one season. Supplementing of WTP water varied by seasonal availability of other water sources and by community (Table 2). Participants from the Interior community supplemented WTP water with other sources more than the Northwest community, except in summer (Fig. 2). Across all seasons, Interior households reported hauling an average (± 1 SD) of 1.1 ± 2.2 gpcd of water from supplemental sources, whereas Northwest homes hauled 0.5 ± 3.4 gpcd of water from supplemental sources.

### Water quality

#### Water quality by source

Water samples were taken from 309 containers across both communities over four seasons. Thirty percent (*n* = 92) of all samples tested positive for total coliforms (mean ± SD = 45 ± 179 MPN/100 mL, 95% confidence interval = 25–65 MPN/100 mL) and 2% (*n* = 5) tested positive for *E. coli*. Twenty-seven percent of 258 WTP samples, 55% of 17 rainwater samples, 20% of 15 river samples, and 60% of 5 snow melt samples tested positive for total coliforms (Table 3). There was a significant difference in total coliform counts (MPN/100 mL) between rainwater (mean ± SD = 81 ± 250) and WTP samples (39 ± 164, *p* = 0.002) and between rainwater and river samples (69 ± 261, *p* = 0.04). There were also significant differences between total coliform counts in summer (mean ± SD = 72 ± 228) and winter (21 ± 125, *p* = 0.05) and between fall (23 ± 99) and winter (*p* = 0.02).

**Table 3.** Total Coliform and *Escherichia coli* Concentrations from Household Stored Water Samples (*N*) in Different Seasons and from Different Water Sources in Two Communities

	Number of samples	Total coliforms		Total coliforms	*E. coli*
		Mean ± SD, MPN/100 mL	95% CI, MPN/100 mL	% positive (*N*)	% positive (*N*)
All samples	309	45 ± 179	25–65	30 (92)	2 (5)
Samples by source					
WTP	258	39 ± 164	19–59	27 (69)	≤1 (2)
Rain	31	81 ± 250	0–172	55 (17)	10 (3)
River	15	69 ± 261	0–213	20 (3)	0 (0)
Snow	5	76 ± 141	0–251	60 (3)	0 (0)
Samples by season					
Winter	66	21 ± 125	0–52	20 (13)	0 (0)
Spring	70	58 ± 215	7–109	27 (19)	0 (0)
Summer	92	72 ± 228	5–94	33 (30)	5 (5)
Fall	81	23 ± 99	1–45	37 (30)	0% (0)

#### Water quality by storage

Household water was most commonly stored in 10- to 50-gallon plastic trashcans (*n* = 100), filtered water dispensers or pitchers (*n* = 98), jerry jugs (*n* = 35), sealed water tanks (*n* = 35), and 5-gallon buckets (*n* = 22). Containers ranged in size from 0.5 to 200 gallons (mean = 26 gallons, median = 6 gallons). Of 325 water containers sampled, 77% (*n* = 251) were fully covered and 15% (*n* = 50) were uncovered or partially covered (7%, *n* = 24 had no information). There was no significant difference in total coliform counts between covered (mean ± SD = 35.2 ± 154.3) and uncovered containers (73.5 ± 249.0, *p* = 0.56). There was a significant difference in total coliform counts between filtered water dispensers or pitchers (mean ± SD = 19.0 ± 110.1) and the trashcans that were most commonly the source water for the filtered water dispensers and pitchers (87.8 ± 255.5, *p* = 0.0004).

### Water reuse

Household water was reused without treatment most commonly for handwashing and laundry, with fewer homes reusing bathwater (e.g., for multiple children before disposing, *n* = 8 homes, 20% of respondents) or dishwater (e.g., by using the same water throughout the day, *n* = 27 homes, 68%). Of 40 households in both communities, 90% (*n* = 36) of households indicated using a wash basin for handwashing, where soap (when used) is mixed with standing water in a basin to wash hands. Two homes (5%) had created flow-through washing systems with hauled water to avoid wash basin use, and two homes (5%) said they did not wash their hands. Households that used wash basins reported reusing the same water an average of three times before dumping it out. Wash basin reuse ranged from 0 to 18 times, with 80% of interviews indicating reusing at least once.

Eighteen households reported reusing laundry water at least once (78% of the 23 households that washed laundry at home), and laundry water was reused for 1–7 loads. Most homes indicated that they determine reuse by how dirty they perceived the clothes to be before washing and how dirty the water was afterward. Several households also volunteered accounts of conserving the amount of water used for laundry by only using a single batch of water for washing laundry, instead of using two batches: one for washing and one for rinsing the soap and dirt off the clothes.

### Waste management

#### Greywater disposal

To dispose of used wash water from dishes, handwashing, bathing, laundry, and cleaning (collectively “greywater”), households reported manually dumping the water outside, draining it through gravity pipes that discharged into the open space located directly underneath the home (if the home had a raised foundation), or dumping it underground (into a soak pit in the Northwest or outhouse toilet in the Interior). Discharging underground is the safest behavior to reduce subsequent exposure to waste, which was performed in 7–13% of households, depending on the season. Underground disposal was achieved using hand-dug soak pits or disposing greywater into pit latrines. Discharging >5 m away from the home was practiced in 10–25% of homes, depending on the season. Discharging <5 m away from the home or directly underneath the home on the ground surface was practiced in 68–79% of homes (Table 4).

**Table 4.** Greywater Disposal Locations from Unpiped Households (n) in Two Communities by Season

No. of households	≤5 m away from home or underneath home	≥5 m away from home	Underground in pit or outhouse
Fall (*N* = 30)	23	3	4
Winter (*N* = 28)	19	7	2
Spring (*N* = 29)	21	5	3
Summer (*N* = 34)	27	4	3

#### Human waste disposal

**FIG. 3.**
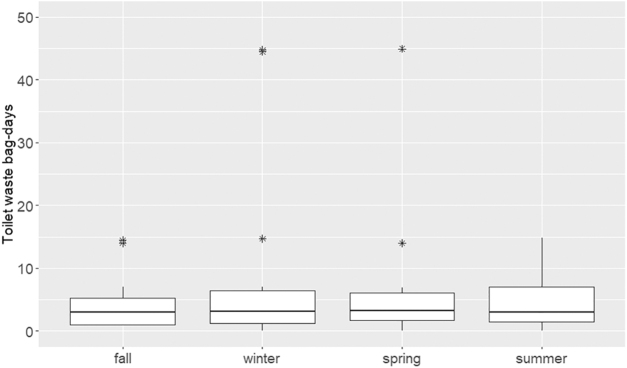
Number of bag-days (no. of toilet bags sitting outside times number of days) that human waste sits outside of the home before being hauled away for disposal. Results are self-reported from households using bucket latrines (“honeybuckets”) in two communities by season. Y-axis is truncated and does not display highest *y*-values. Boxes represent interquartile range (IQR). Horizontal mid-line represents median value. Vertical lines show values less than 1.5 times the IQR. Asterisks denote outliers outside of 1.5 times the IQR above the upper quartile and below the lower quartile.

All participating households in the Interior community used outhouse pit latrines as their primary toilet, whereas homes in the Northwest used bucket latrines inside the home, locally called “honeybuckets.” Some Interior households reported using honeybuckets as secondary toilets as well, mostly for elders or young children at night or during periods of extreme weather. Honeybuckets were typically lined with plastic bags before use, and human waste-filled bags were removed from the buckets every 0.3 days/resident on average (range = 0.1–5.5 days per resident). Honeybucket bags were stored outside in plastic or cardboard boxes for 13 days on average (range = 0–365 days) before being hauled to the outhouse (in the Interior) or to the community dumpsite (in the Northwest). Based on the number of times a day honeybuckets were emptied and the frequency of hauling to a final disposal site, human waste was calculated to be sitting outside the home for an average of 4.0 bag-days in fall, 7.1 bag-days in winter, 12.9 bag-days in spring, and 5.13 bag-days in summer (Fig. 3; one “bag-day” is one honeybucket bag sitting outside for 1 day). Although over half of households dispose of their waste within 1 week, some human waste remains near homes for several months to a year before it is hauled away. A few households volunteered that bags would sometimes break while they were sitting outside and leak into the surrounding soil, prompting concerns that feces was getting into airborne dust and into puddles, drainages, and waterways during the spring melt. Several households also mentioned concerns of dogs or birds (ravens) getting into honeybucket bags and spreading fecal matter through the community. However, we were unable to quantify these occurrences.

## Discussion

### Variation in WASH practices by community and household

Heterogeneity in seasonal WASH practices was anticipated based on studies of seasonal water sources in other communities (e.g., Hadjer *et al.*, 2005; Pearson *et al.*, 2016; Elliott *et al.*, 2017), because of the extreme seasonal weather, daylight, and subsistence patterns in rural high-latitude communities (McNeeley and Shulski, 2011), and because of changes in access to and reliability of community infrastructure. Many respondents in this study offered context and comparison between seasons in each interview to describe why certain practices varied in frequency in different seasons or how decisions about water and waste were made. However, the aggregated data presented here demonstrate a high amount of variation within households and communities that obscures some seasonal trends.

Seasonal variation in water quantity used was attributed by some interviewees to changing needs based on household and subsistence activities, transportation access, and weather. For example, some households indicated that water hauling was easier in winter because they were able to drag a sled instead of hand-carrying water buckets, but some households also said that they hauled less in winter because personal bathing and house cleaning needs were lower than in warmer seasons. Although some homes indicated having to haul more water in the summer to wash fish from subsistence activities, others explained that they washed fish at the river or at fish camp and did not need to haul any water to the home for that activity. The variation by season in water access, water haul, and water use practices are consistent with the highly seasonal lifestyle of residents in these communities and could be indicative of changing household water requirements throughout the year. The high heterogeneity in specific household preferences should be considered when trying to apply broad formulas to the design of water and sanitation infrastructure. A longer study of more households in different communities within each region may further illuminate seasonal variation in water practices.

### Water access for health

Water quantity hauled to the home ranged from 3.0 to 5.4 gpcd depending on the community and season, which is consistent with general estimates of water quantity used by self-haul households (Howard and Bartram, 2003), but higher than several previous estimates for rural Alaskan households (Eichelberger, 2010, 2017; Thomas *et al.*, 2016). This study is the first to our knowledge that examines seasonal variation in household water quantity hauled in rural Alaska and attempts to quantify supplemental sources of water from untreated sources. Our results indicate that natural sources offer an average additional 0.7 gpcd (13–23%) to household water use, and only rainwater contains statistically higher numbers of bacteria than stored samples from treated water sources. Additional promotion of and access to untreated water sources could reduce the burden of hauling water from a centralized treatment point (e.g., the use of on-site rain or snow resources; Mattos *et al.*, 2019) and could allow households to access water at convenient times, instead of only being able to get water for a few hours a day when the watering point is open. Overall, 30% of all household-stored water samples from all sources tested positive for total coliforms, suggesting that safe transport, safe storage, hygienic access (e.g., clean dippers), and/or point-of-use water treatment may be needed to improve household-stored water quality, especially for certain water sources (e.g., rainwater) and during certain times of year (e.g., summer). Only 2% of all samples tested positive for *E. coli*, so water quality in the home can generally be considered low risk (World Health Organization, 2011).

The average quantities recorded in this study represent 57–100% (depending on season) of the water quantity defined by the World Health Organization (WHO) for basic access, but only 23–41% of the water quantity recommended for intermediate access (Howard and Bartram, 2003). Because households report using water for consumption, hygiene, and household uses, they are likely not meeting WHO recommendations for any of those categories. (For example, households in this study reported drinking <0.5 gpcd on average.) Accessing enough water in the home to perform personal hygiene activities and prevent water-washed diseases, such as skin, respiratory, and gastrointestinal infections (Webber, 2004), is a major challenge for unpiped communities (Gessner, 2008; Eichelberger, 2010; Wenger *et al.*, 2010; Thomas *et al.*, 2016). The promotion of good quality, untreated natural water sources that are more convenient, such as rainwater catchment (Mattos *et al.*, 2019) or snow melt, alongside point-of-use treatment technologies, could expand the amount of water available in the home and reduce vulnerability to environmental variation (Daley *et al.*, 2014), such as climate change (Elliott *et al.*, 2017) or damages to water infrastructure.

### Expanded pathways of pathogen exposure

WASH infrastructure is often thought to mainly impact fecal–oral routes of disease exposure; however, the WASH practices in the unpiped communities described here suggest four major concerns related to pathogen exposure—low water use in the home, untreated water reuse, inadequate greywater disposal, and inadequate human waste disposal.

#### Low water use in the home

Low water use inhibits removal of pathogens from the body and can increase the likelihood of transferring respiratory, skin, or fecal pathogens through direct contact, floors, or surfaces (Webber, 2004), especially in overcrowded shared spaces (Daley *et al.*, 2014; Singleton *et al.*, 2016). Water use is primarily increased by the provision of piped water systems (Howard and Bartram, 2003; Overbo *et al.*, 2016); however, alternative actions, such as increasing household water storage capacity, which had a significant correlation to quantity of water hauled, or encouraging rainwater catchment during appropriate times of year, can also contribute to higher daily per capita water use.

#### Untreated water reuse

Unpiped households appear to create hierarchies of water management that include reducing the amount of water used for specific activities and directly reusing water, especially for handwashing. Whereas handwash water is sometimes considered “light greywater” (Oktor and Çelik, 2019), it can still contain high numbers of total coliforms, *E. coli* (1.8–7.4 log_10_ CFU/100 mL and 0–3.7 log_10_ CFU/100 mL, respectively; Ottoson and Stenström, 2003) and skin bacteria (Keely *et al.*, 2015). Although it is well-known that handwashing improves health (e.g., Curtis *et al.*, 2000; Kumar *et al.*, 2017; Navab-Daneshmand *et al.*, 2018), it is not clear whether the approach of reusing untreated greywater in basins for handwashing is going to do more good than harm. Similarly, laundry water has been shown to have high levels of total coliforms, *E. coli* (1.9–5.9 log_10_ CFU/100 mL and 0–5.4 log_10_ CFU/100 mL, respectively; Ottoson and Stenström, 2003) and *Staphylococcus aureus* (Tom Hennessy, unpublished data), which leads to a higher exposure risk when laundry water is reused between loads. Untreated reuse can be addressed in the home by providing access to more water for hygiene purposes, including untreated raw sources that have better microbial quality than reused water, and by providing infrastructure that encourages single-use behaviors, such as flow-through sinks instead of wash basins.

#### Greywater disposal

Unpiped households do not often have a safe option for the disposal of greywater, especially in cold climates where any system created to dispose off greywater underground or away from the home is prone to freeze issues. Local soil conditions (e.g., permeability, permafrost presence) also impact the options available to households. Although not directly asked in our interviews, some interviewees in this study expressed the belief that greywater is not a high exposure risk for pathogens, commonly saying about their disposal process, “It's just greywater. There's no poop in it.” This is a common misconception, despite research showing that pathogens and fecal indicators such as *Pseudomonas aeruginosa* (Casanova *et al.*, 2001a), fecal coliforms (Casanova *et al.*, 2001b; Winward *et al.*, 2008), *S. aureus* (Winward *et al.*, 2008), *Legionella, Cryptosporidium,* and *Giardia* (Birks *et al.*, 2004) are present in greywater, sometimes in concentrations comparable with raw wastewater (Katukiza *et al.*, 2015). Greywater in lower income areas, such as the study communities described here, has been shown to be more concentrated because of low water use (Katukiza *et al.*, 2015), and when this greywater is disposed on the ground it has been shown to contaminate areas with fecal coliforms (Casanova *et al.*, 2001b). Direct contact with greywater during disposal or subsequently through activities near the disposal site, such as through boot or vehicle tire contamination (as shown with fecal matter in Chambers *et al.*, 2009) or subsistence activities (Daley *et al.*, 2018), has been characterized as a significant risk for health (Ottoson and Stenström, 2003; Benami *et al.*, 2016). Furthermore, greywater pathogens can become airborne attached to dust or air particles, resulting in additional exposure pathways. Appropriate disposal methods for greywater where risk of human contact is diminished is critical for reducing pathogen exposure in unpiped communities.

#### Human waste disposal

Honeybuckets pose inherent challenges for waste management. The combination of urine and feces in a bucket latrine increases the total volume of highly contaminated waste to dispose (e.g., compared with a urine-diverting toilet Gunnarsdóttir *et al.*, 2013). Furthermore, this study provides evidence that challenges with transportation at different times of year can lead to toilet bags sitting outside for an average of 4–13 days, depending on the season. Weather patterns also challenge good waste management practices, for example, when snowdrifts cover up waiting toilet bags for months until spring thaw reveals them, making it difficult for households to haul as frequently in the winter. The longer human waste sits outside the home, the more likely bags are to get ripped open by the elements or animals, the more likely waste is to spread onto nearby roads and waterways, the more likely pathogens are to attach to dust particles and become airborne, and the more likely humans, especially children, are to come into contact with their contents.

**FIG. 4.**
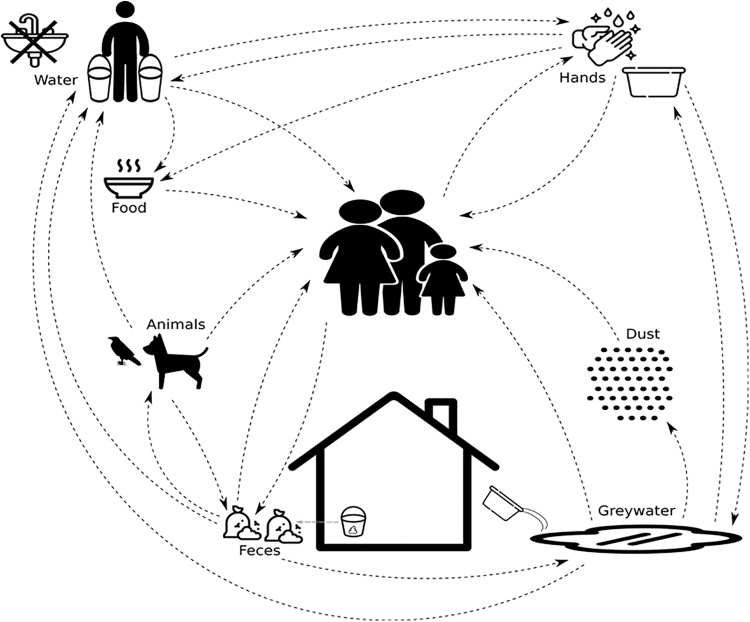
A model of pathogen exposure describing multiple transmission routes identified in this study. This model can be used to implement targeted WASH interventions that can efficiently improve community health and wellbeing.

These concerns can be mapped onto a diagram that illuminates high priority pathways of disease transmission in unpiped communities (Fig. 4) and that can provide a targeted model for WASH interventions that are needed in specific contexts. Figure 4 demonstrates that the lack of access to water for hygiene purposes can allow fecal and nonfecal pathogens, such as from respiratory droplets or skin, to move through the environment to new hosts. Untreated water reuse and inadequate greywater disposal allows for re-exposure of hosts to pathogens directly through contact with dirty water or indirectly when greywater pathogens get picked up in road dust or contaminate surfaces. The presence of animals without adequate waste management and access to water for hygiene purposes can further amplify pathogen spread.

### Recommendations for WASH improvements

Remote Alaska Native communities contend with a variety of challenges related to WASH infrastructure including high construction costs, high energy needs and costs, aging infrastructure, and extreme climates. These are being compounded by a rapidly changing environment and a widening gap between the cost of development projects and the funding available for such projects (Alaska Department of Environmental Conservation, 2017). In addition to the well-known health impacts of fecal–oral transmission of disease, this study has identified several additional routes of pathogen exposure from household and community WASH practices that need to be addressed to reduce the incidence of water-washed disease. We further provide information to support interim actions that can be undertaken by individuals, tribes, and regional/state/national health organizations, that are less expensive and less permanent than piped infrastructure, but that can still build community resilience and improve health:

- Improve water storage capacity and safe storage. Households with larger storage capacities tend to use more water and can also haul less often and worry less about rationing or running out of water.
- Increase access to natural water resources to supplement treated water. Houses with the capability of catching rainwater during rainy seasons have reported lower burdens of hauling water, and many elders prefer certain traditional water sources because of cultural connections.
- Increase access to flow-through water for handwashing through simple household infrastructure. Households that have been able to hook up simple plumbing systems decrease contamination of water and reported increased water use for certain activities (e.g., showers and handwashing).
- Improve greywater knowledge and handling practices. Many individuals believe that greywater is harmless compared with fecal matter, resulting in surface disposal and high risk of pathogen transmission through multiple pathways throughout the community.
- Develop community-wide waste hauling systems. Interviewees cited significant challenges for managing their waste individually, including lack of transportation. A community haul system would reduce everyone's risk of exposure to fecal matter.

### Limitations

This study examined WASH practices in 40–50% of households in two unpiped communities in two different regions of Alaska through seasonal single point-in-time interviews. The data collected here may not be fully representative of all communities nor of household practices throughout the year or across years. We relied on self-reported practices that required interviewees to be aware of their behaviors and comfortable reporting them truthfully to interviewers. Furthermore, multiple measures of water quantity and quality were collected, but only selected measured are reported here. Limited qualitative data were discussed here, and conclusions would be strengthened by a qualitative analytical framework. Additional data collected from more households representing other communities, regions, and countries would add to the weight of this dataset.

## Summary

Improving health and wellbeing through WASH is a global priority; however, needs vary greatly between countries, regions and communities, and can vary within communities by season or household. This study summarizes practices related to water quantity and sources hauled to the home, household water quality, informal water reuse, and greywater and toilet waste disposal in two remote, rural, unpiped communities. We further evaluate critical under-discussed pathways of pathogen exposure based on reported household WASH practices and provide recommendations for intermediate actions that can be taken to reduce exposure and improve health while communities pursue piped infrastructure. This model of examining specific household practices to determine transmission routes can be applied to other remote communities or to unique conditions to aid in the recommendation of targeted WASH interventions.
